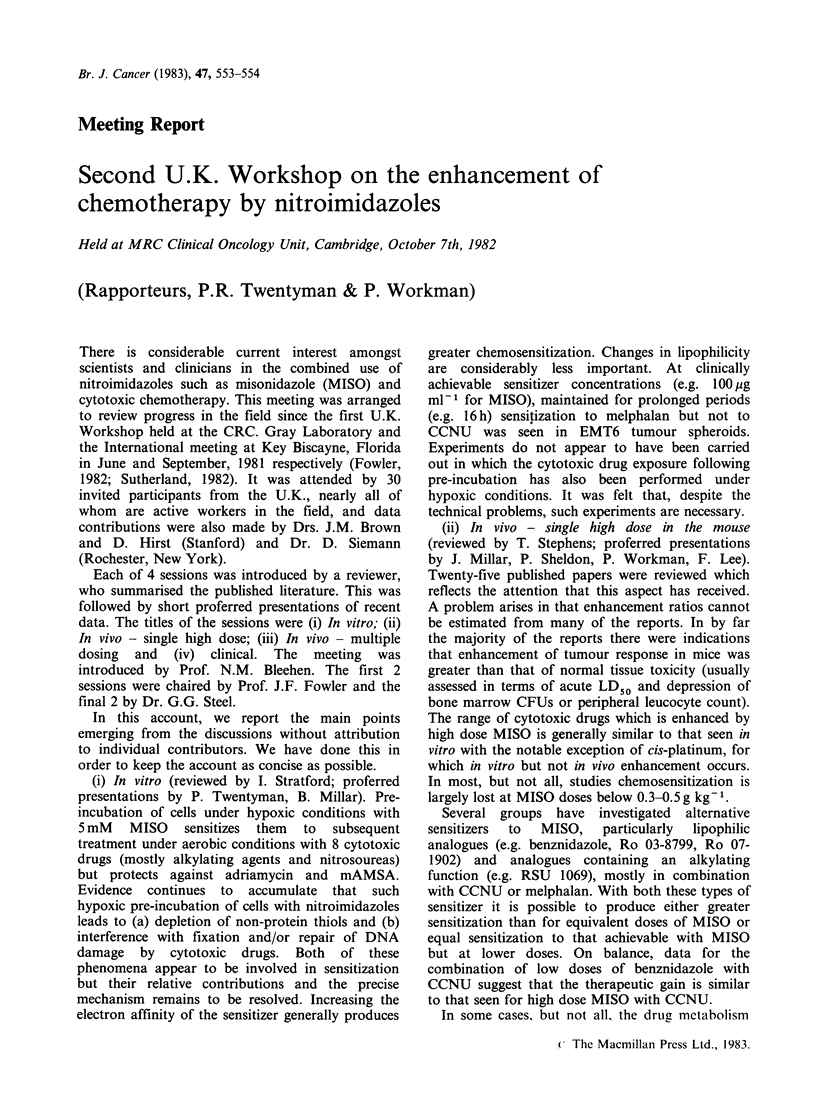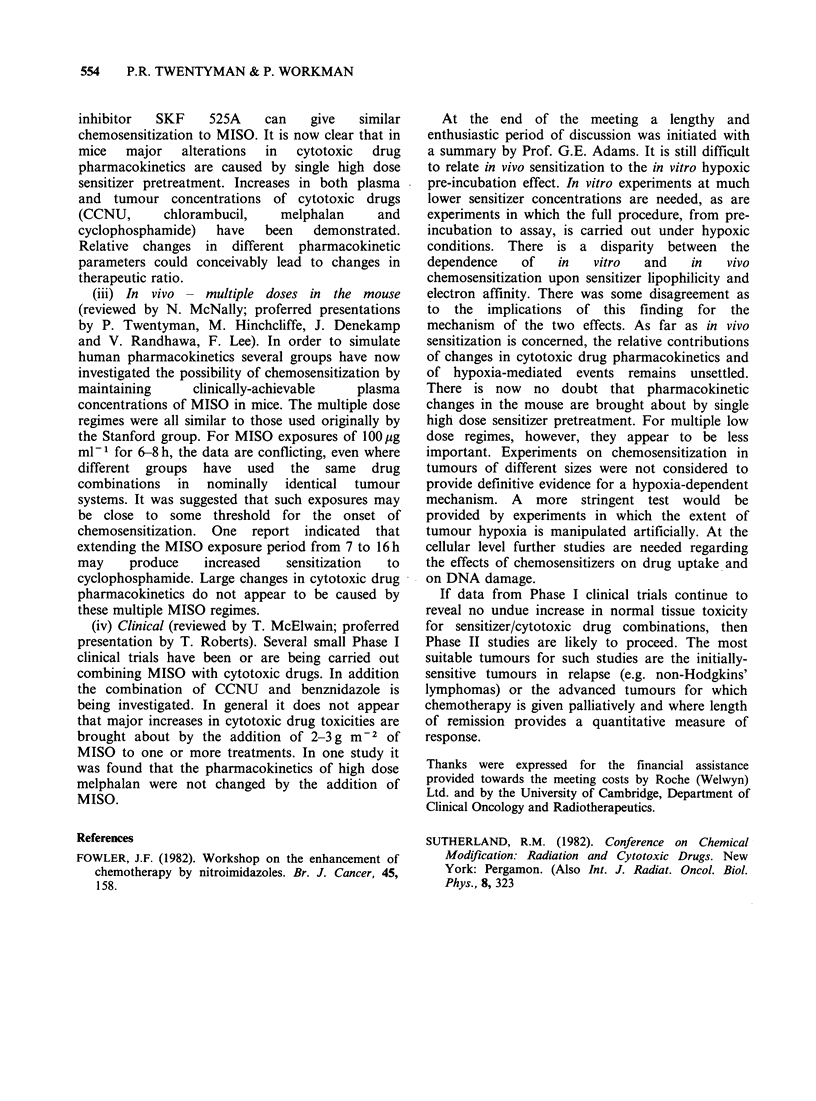# Second U.K. workshop on the enhancement of chemotherapy by nitroimidazoles

**Published:** 1983-04

**Authors:** 


*Br. J. Cancer* (1983), **47**, 553–554

### Meeting Report

### Second U.K. Workshop on the enhancement of chemotherapy by nitroimidazoles

*Held at MRC Clinical Oncology Unit, Cambridge, October 7th, 1982*

(Rapporteurs, P.R. Twentyman & P. Workman)

There is considerable current interest amongst scientists and clinicians in the combined use of nitroimidazoles such as misonidazole (MISO) and cytotoxic chemotherapy. This meeting was arranged to review progress in the field since the first U.K. Workshop held at the CRC. Gray Laboratory and the International meeting at Key Biscayne, Florida in June and September, 1981 respectively (Fowler, 1982; Sutherland, 1982). It was attended by 30 invited participants from the U.K., nearly all of whom are active workers in the field, and data contributions were also made by Drs. J.M. Brown and D. Hirst (Stanford) and Dr. D. Siemann (Rochester, New York).

Each of 4 sessions was introduced by a reviewer, who summarised the published literature. This was followed by short proferred presentations of recent data. The titles of the sessions were (i) *In vitro;* (ii) *In vivo* – single high dose; (iii) *In vivo* – multiple dosing and (iv) clinical. The meeting was introduced by Prof. N.M. Bleehen. The first 2 sessions were chaired by Prof. J.F. Fowler and the final 2 by Dr. G.G. Steel.

In this account, we report the main points emerging from the discussions without attribution to individual contributors. We have done this in order to keep the account as concise as possible.

(i) *In vitro* (reviewed by I. Stratford; proferred presentations by P. Twentyman, B. Millar). Pre-incubation of cells under hypoxic conditions with 5 mM MISO sensitizes them to subsequent treatment under aerobic conditions with 8 cytotoxic drugs (mostly alkylating agents and nitrosoureas) but protects against adriamycin and mAMSA. Evidence continues to accumulate that such hypoxic pre-incubation of cells with nitroimidazoles leads to (a) depletion of non-protein thiols and (b) interference with fixation and/or repair of DNA damage by cytotoxic drugs. Both of these phenomena appear to be involved in sensitization but their relative contributions and the precise mechanism remains to be resolved. Increasing the electron affinity of the sensitizer generally produces greater chemosensitization. Changes in lipophilicity are considerably less important. At clinically achievable sensitizer concentrations (e.g. 100 $\mu$g ml$^{-1}$ for MISO), maintained for prolonged periods (e.g. 16 h) sensitization to melphalan but not to CCNU was seen in EMT6 tumour spheroids. Experiments do not appear to have been carried out in which the cytotoxic drug exposure following pre-incubation has also been performed under hypoxic conditions. It was felt that, despite the technical problems, such experiments are necessary.

(ii) *In vivo – single high dose in the mouse* (reviewed by T. Stephens; proferred presentations by J. Millar, P. Sheldon, P. Workman, F. Lee). Twenty-five published papers were reviewed which reflects the attention that this aspect has received. A problem arises in that enhancement ratios cannot be estimated from many of the reports. In by far the majority of the reports there were indications that enhancement of tumour response in mice was greater than that of normal tissue toxicity (usually assessed in terms of acute $LD_{50}$ and depression of bone marrow CFUs or peripheral leucocyte count). The range of cytotoxic drugs which is enhanced by high dose MISO is generally similar to that seen *in vitro* with the notable exception of *cis*-platinum, for which *in vitro* but not *in vivo* enhancement occurs. In most, but not all, studies chemosensitization is largely lost at MISO doses below 0.3–0.5 g kg$^{-1}$.

Several groups have investigated alternative sensitizers to MISO, particularly lipophilic analogues (e.g. benznidazole, Ro 03-8799, Ro 07-1902) and analogues containing an alkylating function (e.g. RSU 1069), mostly in combination with CCNU or melphalan. With both these types of sensitizer it is possible to produce either greater sensitization than for equivalent doses of MISO or equal sensitization to that achievable with MISO but at lower doses. On balance, data for the combination of low doses of benznidazole with CCNU suggest that the therapeutic gain is similar to that seen for high dose MISO with CCNU.

In some cases, but not all, the drug metabolism

© The Macmillan Press Ltd., 1983.

inhibitor SKF 525A can give similar chemosensitization to MISO. It is now clear that in mice major alterations in cytotoxic drug pharmacokinetics are caused by single high dose sensitizer pretreatment. Increases in both plasma and tumour concentrations of cytotoxic drugs (CCNU, chlorambucil, melphalan and cyclophosphamide) have been demonstrated. Relative changes in different pharmacokinetic parameters could conceivably lead to changes in therapeutic ratio.

(iii) *In vivo – multiple doses in the mouse* (reviewed by N. McNally; proferred presentations by P. Twentyman, M. Hinchcliffe, J. Denekamp and V. Randhawa, F. Lee). In order to simulate human pharmacokinetics several groups have now investigated the possibility of chemosensitization by maintaining clinically-achievable plasma concentrations of MISO in mice. The multiple dose regimes were all similar to those used originally by the Stanford group. For MISO exposures of 100 $\mu$g ml$^{-1}$ for 6–8 h, the data are conflicting, even where different groups have used the same drug combinations in nominally identical tumour systems. It was suggested that such exposures may be close to some threshold for the onset of chemosensitization. One report indicated that extending the MISO exposure period from 7 to 16 h may produce increased sensitization to cyclophosphamide. Large changes in cytotoxic drug pharmacokinetics do not appear to be caused by these multiple MISO regimes.

(iv) *Clinical* (reviewed by T. McElwain; proferred presentation by T. Roberts). Several small Phase I clinical trials have been or are being carried out combining MISO with cytotoxic drugs. In addition the combination of CCNU and benznidazole is being investigated. In general it does not appear that major increases in cytotoxic drug toxicities are brought about by the addition of 2–3 g m$^{-2}$ of MISO to one or more treatments. In one study it was found that the pharmacokinetics of high dose melphalan were not changed by the addition of MISO.

At the end of the meeting a lengthy and enthusiastic period of discussion was initiated with a summary by Prof. G.E. Adams. It is still difficult to relate *in vivo* sensitization to the *in vitro* hypoxic pre-incubation effect. *In vitro* experiments at much lower sensitizer concentrations are needed, as are experiments in which the full procedure, from pre-incubation to assay, is carried out under hypoxic conditions. There is a disparity between the dependence of *in vitro* and *in vivo* chemosensitization upon sensitizer lipophilicity and electron affinity. There was some disagreement as to the implications of this finding for the mechanism of the two effects. As far as *in vivo* sensitization is concerned, the relative contributions of changes in cytotoxic drug pharmacokinetics and of hypoxia-mediated events remains unsettled. There is now no doubt that pharmacokinetic changes in the mouse are brought about by single high dose sensitizer pretreatment. For multiple low dose regimes, however, they appear to be less important. Experiments on chemosensitization in tumours of different sizes were not considered to provide definitive evidence for a hypoxia-dependent mechanism. A more stringent test would be provided by experiments in which the extent of tumour hypoxia is manipulated artificially. At the cellular level further studies are needed regarding the effects of chemosensitizers on drug uptake and on DNA damage.

If data from Phase I clinical trials continue to reveal no undue increase in normal tissue toxicity for sensitizer/cytotoxic drug combinations, then Phase II studies are likely to proceed. The most suitable tumours for such studies are the initially-sensitive tumours in relapse (e.g. non-Hodgkins' lymphomas) or the advanced tumours for which chemotherapy is given palliatively and where length of remission provides a quantitative measure of response.

Thanks were expressed for the financial assistance provided towards the meeting costs by Roche (Welwyn) Ltd. and by the University of Cambridge, Department of Clinical Oncology and Radiotherapeutics.

### References

FOWLER, J.F. (1982). Workshop on the enhancement of chemotherapy by nitroimidazoles. *Br. J. Cancer*, **45**, 158.

SUTHERLAND, R.M. (1982). *Conference on Chemical Modification: Radiation and Cytotoxic Drugs*. New York: Pergamon. (Also *Int. J. Radiat. Oncol. Biol. Phys.*, **8**, 323